# Virtual reality for biochemistry education: the cellular factory

**DOI:** 10.1007/s10639-023-11826-1

**Published:** 2023-05-13

**Authors:** John Barrow, William Hurst, Joakim Edman, Natasja Ariesen, Caspar Krampe

**Affiliations:** 1 Institute of Medical Sciences, University of Aberdeen, Aberdeen, AB25 2ZD UK; 2 Information Technology Group, Wageningen University and Research, Leeuwenborch, Hollandseweg 1, 6706 KN Wageningen, the Netherlands; 3 Marketing and Consumer Behaviour Group, Wageningen University and Research, Leeuwenborch, Hollandseweg 1, 6706 KN Wageningen, the Netherlands

**Keywords:** Immersive Learning, Virtual Reality, Electrodermal Activity, Pedagogy, Biochemistry

## Abstract

Virtual Reality (VR) involves the coupling of visual communication hardware and software. The technology is capable of offering transformative educational practice and is increasingly being adopted within the biochemistry domain to better understand complex biochemical processes. This article documents a pilot study for the efficacy of VR in biochemistry education at undergraduate university level, focusing on the citric acid cycle: a central process for extracting energy in most cellular life forms. 10 participants were equipped with a VR headset and electrodermal activity (EDA) sensors, then immersed within a digital environment where they were able to learn the 8 main steps of the citric acid cycle within a virtual lab by completing 8 levels of activity. Post and pre surveys were taken, along with EDA readings throughout the students’ interaction with VR. Research findings support the hypothesis that VR increase students’ understanding, particularly if students feel engaged, stimulated and intend to use the technology. Moreover, EDA analysis indicated that the majority of participants demonstrate enhanced engagement in the education-based VR-experience as measured by elevated levels of skin conductance, a marker for autonomic arousal and a measure of engagement in an activity.

## Introduction

Biochemistry can be defined as the chemical processes that occur in the cells of all cellular life forms. These processes are crucial to all forms of life as the reactions, signalling mechanisms and other cellular processes are required in order for life to function. In this regard they form the basis of many life science curricula. Virtual Reality (VR), as a visual communication tool, has the potential to disrupt the traditional pedagogic practice educators employ when teaching university-level students within this domain. This is because the technology has been proven to help learners retain meaningful information and understand challenging and often abstract theory (Coan et al., 2020) by means of immersion and embodiment within a digital environment. As evidence, work in this area by Kim et al. (2019) details the development of a VR-based visualisation model specifically for biochemistry and Bennie et al. (2019) adopt the technology for teaching enzyme catalysis, thus demonstrating the impact of integrating VR applications into existing educational practices. Other related works, such as Escaping the Cell by Christopoulos et al. (2022) and a VR Molecular Builder by Pietikäinen et al. (2021) expand this notion further through the development of a VR-based escape room for biology education and VRChem for building, visualising and manipulating organic molecules respectively.

The biochemical processes happen at a molecular level thousands of times smaller than the size of the cells in which they occur. Because of this, students often struggle to understand how these processes impact on life, human health and disease, and quite often, are required to understand 3D information that is delivered in a 2D format (Bennie et al., 2019). Virtually all metabolic processes work by the molecules involved interacting in a specific way dictated by the shapes of the molecules involved. Allowing students to visually immerse themselves into physically out of reach processes within a VR-based impossible field trip or virtual field trip setting (Dolphin et al., 2019), offers a metric to let participants understand that the form and the function of the process go hand-in-hand.

All cells contain interlinked series of reactions called biochemical pathways that are dynamic in nature and exist to carry out reactions that allow cells to break down food molecules or create new molecules required for the cells to function—a process known as metabolism. These interlinked metabolic processes are comparable to cells acting like factory production lines. Students learning the processes typically experience four layers of learning: *1)* Basic reaction processes; *2)* Linked sets of reactions arranged in a pathway; *3)* Regulation and control of the pathway; and *4)* Links between pathways.

Yet, students struggle with all aspects of these layers especially as one builds on the other, so not understanding one layer would make it challenging to understand the subsequent layers. This interlinked nature of the subject, and students being able to understand how form and function are combined, make some foundational aspects of biochemistry challenging subjects to teach. Also, metabolic processes are dynamic but often taught using static imagery and diagrams in textbooks and lecture slides.

Therefore, there are two major problems we aim to solve with this research: *1)* students do not always understand how form and function go together, and *2)* that the processes are dynamic and learning structure is hierarchical. We theorise that these two problems can be addressed by means of harnessing the advanced communication potential of VR technologies; continuing towards a shift in the way foundational biochemistry is taught. Thus, an investigation will be conducted into the engagement levels of a group of 10 undergraduate-level university students. We adopt three assessment techniques to measure the change: *1)* the Kirkpatrick & Kirkpatrick evaluation framework (reaction, learning, behaviour, result) that has proven to be effective for the evaluation of training outcomes in varied domains of application (Cahapay, 2021; Smidt et al., 2009); *2)* multiple linear regression analysis to compare the quantitative difference between the pre/post educational experience; *3)* EDA to measure potential physiological arousal (i.e. engagement or excitement).

The remainder of this article is as follows. Section 2 provides a discussion on the background and related work within the domain of VR for biochemistry education. Section 3 outlines the research methodology adopted, with the presentation of the results documented in Section 4. The article is concluded in Section 5.

## Background and related work

The notion of VR-based education is not a new one. For example, in 1997 Hoffman et al. discussed incorporating VR into educational processes due to the technology’s enormous educational potential (Hoffman & Vu, 1997). This belief has gained much traction since 2018 (with the release of the lower cost and untethered Oculus Go), when VR-based hardware started to become more affordable to the average consumer. Further, software development kits (SDK)—supported by Unity and Unreal game engines—are readily accessible for low-cost application development. Yet, at the time of writing this article, research within the domain of VR-based pedagogical applications for biochemistry remains relatively within its infancy and still to be adopted at any meaningful wide-scale. One reason for this could be the lack of knowledge about how VR can be used in the specific context of biochemistry education.

In the following subsection an overview of some of the prominent related works in this domain are discussed. As the examples will demonstrate, applications are typically siloed with their deployments constrained to case studies and prototype deployments. Whilst the work in this article follows suit, the authors envision that the findings presented will lead towards a best practice guide, transferable to other subjects where similar learning layers are involved. Selection of the articles discussed is conducted using a snowballing approach, allowing the authors to select research from a variety of digital libraries including Scopus, PubMed, ResearchGate, Google Scholar, ACM digital library and IEEE to cover a broad domain of literature.

### Related works: VR

The aforementioned article by Christopoulos et al., focuses on wider biology-based education with the application targeted towards upper secondary school education level (Christopoulos et al., 2022). As the authors discuss, higher-order cognitive skills (e.g., critical thinking, metacognition, systematic decision making, evaluative thinking, etc.) are typically best attained by means of hands-on experimentation. Within biochemistry, a hands-on approach is of course not always possible when learning foundational theory, such as the citric acid cycle. Biochemistry education is considered difficult, requiring a significant portion of time, effort, and cognitive ability (Kim et al., 2019). Therefore, educational multimedia applications are often integrated into the classroom environment, including videos, animations, and interactive digital storytelling. For each, a tangible element is missing and VR has the potential to immerse our senses within life-like experiences (Pietikäinen et al., 2021); thus offering this missing hands-on elements through a combination of Head Mounted Displays (HMD), haptics, controllers (palm grasp hold with wrist-based pointing) and sound.

Coan et al., (2020) take advantage of this immersion in the development of a VR-based education tool for teaching lab sessions covering various biological molecules. Their investigation involves the development of a 3D learning environment, in which students are able to proceed through a learning process of identifying different DNA structures, comparing wildtype and mutant collagen molecules, and finally effects of transcription factor interactions with DNA. Students are then quizzed to gauge their level of understanding. Findings indicate that learners strongly agree the VR helped them learn the material in question. In another example, Kim et al. focus on the tricarboxylic acid (TCA) cycle, with medical students as the participants in their evaluation, in which the authors compare how effective VR is compared to traditional biochemistry learning techniques. Results indicate that there is a statistical significance in long-term memory retention for participants learning with VR. Success in memory retention could be due to the engagement created by a hands-on learning process within a virtual environment. Bennie et al. (2019) outline that third-year university students found VR-based learning more engaging than traditional approaches. In their research, the authors employ VR (by means of the HTC Vive HMD) as a tool to present real-time molecular dynamics simulations as a complement to observations made during wet-lab sessions. Findings conclude that use of the VR tool improved the students’ overall sentiment and impression of computational molecular science, in addition to creating a positive effect on perceived learning outcomes.

Another study by Paxinou et al. tested the effectiveness of traditional, video and VR simulation on student’s knowledge (Paxinou et al., 2019). Their 3D virtual biology laboratory scored a 31.15% increase between the pre-test and the post-test, where the conventional and video scored a 15.35% and 20.31% increase respectively. Furthermore, the students who had the VR-lab experience became more capable when handling the photonic microscope than their peers. Additionally, Johnston et al. suggest that VR can have a significant positive impact on students’ understanding and learning. Thus, they created a VR environment with a landing space to overcome sensory overload for first time VR users. Within the landing room the user becomes acquainted with the controls, after which they can enter a cell membrane. Inside the cell users use the hand controller to touch structures to trigger a voice-over and visual overlay for more information. Finally, an illustration is provided by O’Connor et al., who develop a multi-user virtual reality framework to visualise the structures and dynamics of complex molecular structures (O'Connor et al., 2018). They created a framework where up to 6 users could simultaneously manipulate the molecular mechanics. Users preferred the VR experience for three reasons: 1) depth perception, 2) walking around a molecule and 3) the use of both hands to accomplish the tasks, making the learning experience more tangible.

### Related works: AR

The approach to understanding metabolic pathways is, of course, not only limited to VR. The concept of augmented reality (AR), where a physical object is given a digital component, has also been utilized in teaching fundamental biochemistry and it is important to consider the benefits of the use of both technologies. In a key study, Garzon et al. outline the creation of a modular AR program, Augmented Reality Metabolic Pathways (ARMET), to teach the processes of the Krebs cycle and steroid hormone synthesis (Garzón et al., 2017). They tested their application to visualise the 3D structures on 88 student groups and observed students developed the necessary visual literacy skills with use of the app. Results highlight the ability to collect large datasets on student performance based on worksheets returned after the activity. Only relying on student feedback, the learning outcomes of the ARMET framework, namely interpreting molecular models and metabolic maps, were deemed meaningful. In addition, the study concluded that, not only did AR provide a better approach to understanding abstract concepts; it also provided a key platform to initiate academic debate and visual literacy skills.

Similarly, Williams et al. developed an AR tool to explore protein structures for biochemical and biotechnology education. In total 54 students used AR to project the 3D structure of the sfGFP (superfolder green fluorescent protein) on their benches (Williams, 2020). However, no assessment is conducted on the structure, so the student learning impact is not reported for this specific AR tool. A recent 2021 study created 3D models for four protein crystal structures to be used in the classroom with 20 students participating (Reeves et al., 2021). Students could investigate the 3D protein features such as its subunits, domains, and functional groups. Most of the students indicated that AR had helped them to understand the topics and agreed that AR made the content more engaging.

It is clear that AR and VR rely on similar methods to enhance visual perception. The comparison between AR and VR response in science teaching, as outlined by Huang et al. (2019) suggests that VR remains an ideal approach when considering the two due to the immersion and engagement offered through the mechanism of spatial presence; and thus, the technology is the focus of our own approach. To assess the modality of the information retained the authors set up a VR vs AR environment, focusing on retention of visual and auditory retention. The results confirmed that the VR condition had a stronger cognitive and psychological response to the material compared to AR. Furthermore, the study provides a key insight in the modality of information retained best. Reporting that in combination with increased spatial presence, VR shows stronger retention of visual information than AR. Comparatively, AR provided a better retention of auditory information. These findings support our approach in teaching the citric acid cycle, where focus has been placed on visualisation of the proteins and reactants.

## Methodology

The mixed-methodology employed in this article is comprised of three stages: 1) the design and development of a VR-based educational environment; 2) the testing of the environment and collection of data from university-level test subjects and 3) the evaluation of the collated datasets to document insight into the effectiveness of the VR-based approach. As such, this section is divided into subsections aligning to these three stages.

### VR approach

The development is based on the citric acid cycle. Within the VR environment, each of the citric acid cycle reactions were presented as different levels, resulting in eight levels for the participants to complete. The VR approach comprised haptics, user interface design and software development.

A. *Haptics*

**Fig. 1:**
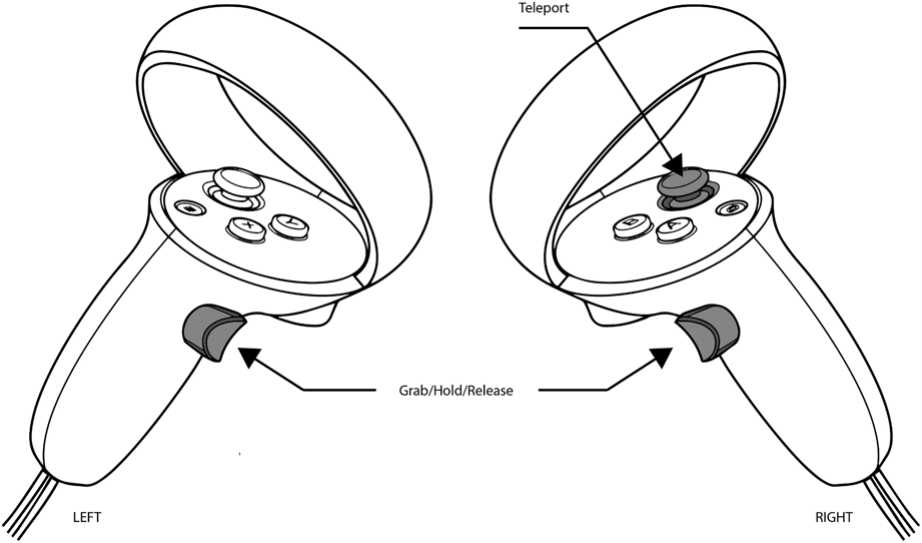
Handheld Controller Setup

The VR environment was deployed on a MetaQuest2 headset for portability, allowing testing to take place without the need for a computer and tether. Interaction with the virtual environment takes place by use of handheld controllers (Fig. 1), employing the palm grasp hold and trigger button press approach for picking up and moving molecular structures and to keep game play as simple, natural, and intuitive as possible.

Movement of the player is via two modes, through joystick teleportation and physical movement of the player. Joystick teleportation is ideal for playing the game in confined spaces or with a stationary-play boundary setup on the Quest2 headset, or if there is space to have a larger-scale play boundary the learner can physically walk around the environment. Both ways of moving reduce the risk of nausea when in the VR environment, giving the user as much control over their virtual movement choices as possible.

B. *User interface and sound*

**Fig. 2:**
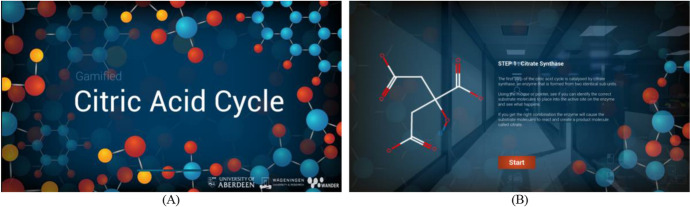
**A**: User Interface/Viewport and **B**: Text concerning Level 1 of the Citric Acid Cycle game

A simplistic user interface was adopted to avoid overload of information. A central reference point was placed in the middle of the viewport to direct the users’ gaze. This also serves as a guide for the selection, pick up and placement of molecules. On entering the virtual world, the visual set up consists of a splash screen that houses text explaining each reaction of the citric acid cycle. A sample of the text is demonstrated in Fig. 2B. The text changes for each level in the citric acid cycle. Sound effects are also included into the environment with calming background music to reduce stress levels and increase the feeling of immersion.

III. *Software development*

**Fig. 3:**
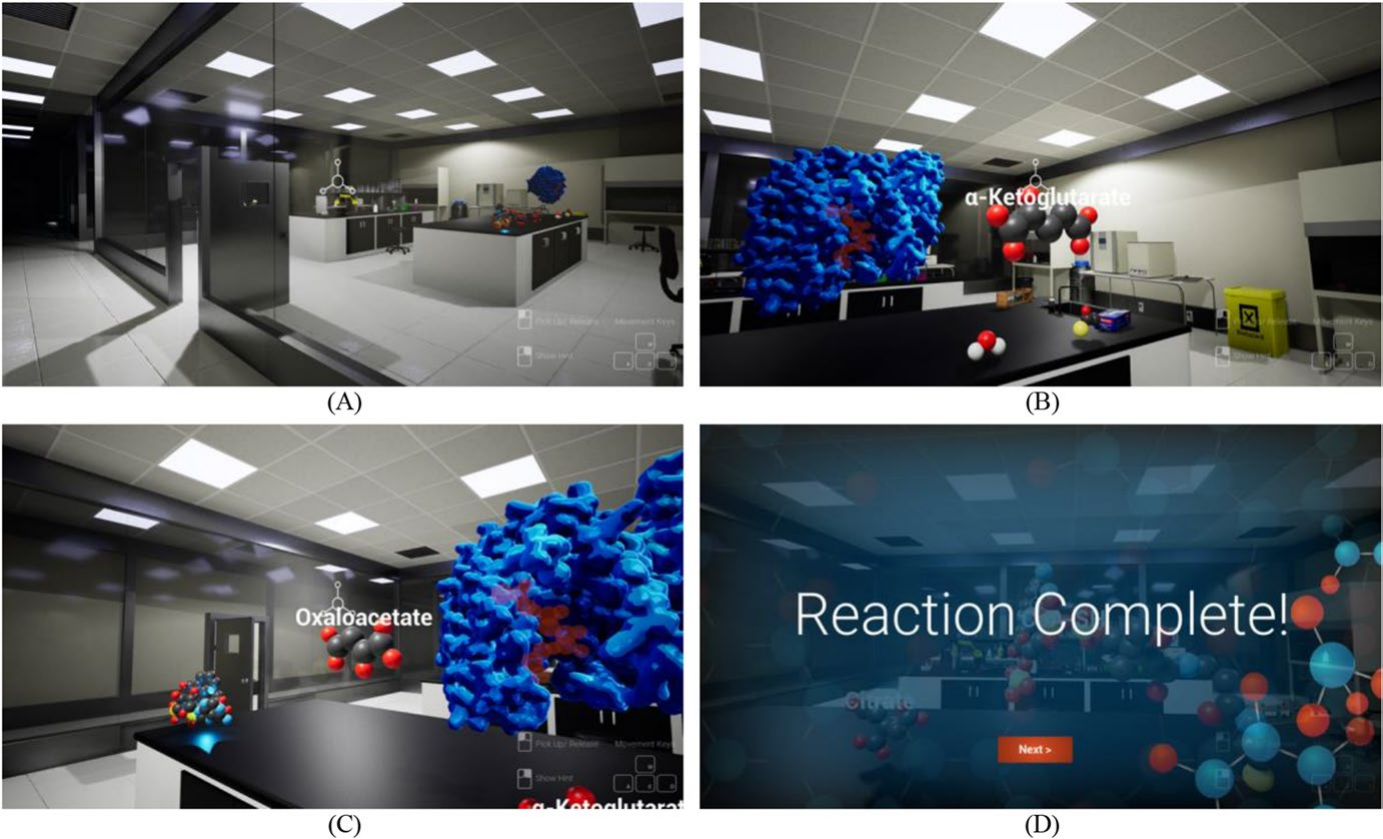
Virtual Environment and Processes. **A**) Lab overview; **B**) & **C**) View of molecules in lab; **D**) Successful completion of Stage 1 Message

**Fig. 4:**
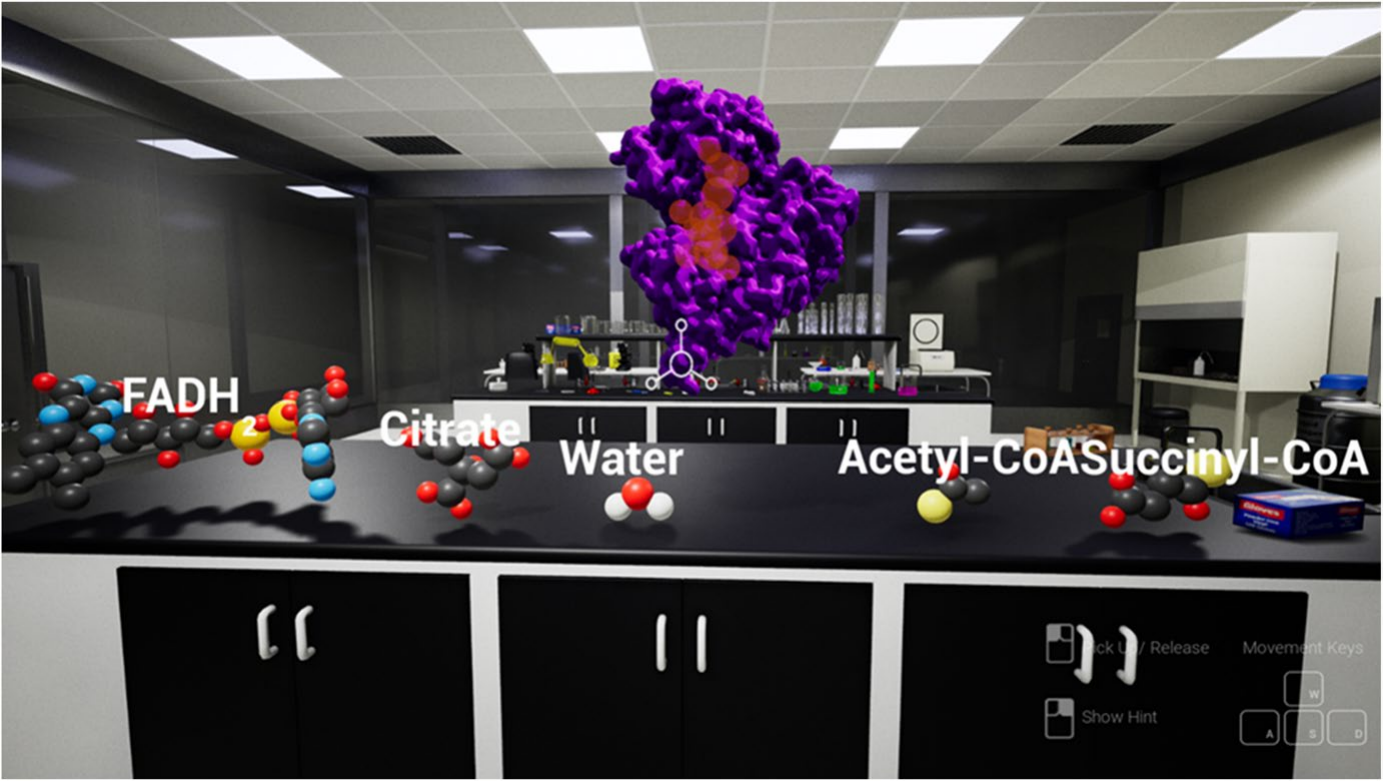
Level 2: Reaction 2 of the Citric Acid Cycle

Within the VR environment, the user is presented with a laboratory environment (Fig. 3A.), in which the user can freely walk around. This freedom of movement is an ideal metric to reduce the level of nausea when working in a VR environment (Coan et al., 2020). Collision is placed on static game assets such as walls, desks, chairs, etc., to cater for realism. Lighting is only provided on one section of the environment: the room in which the citric acid cycle molecules are present (Fig. 3B and C). This lighting effect draws the user’s attention to the correct location for the serious play. Figure 4. displays the start of reaction 2 of the citric acid cycle. The development took place within the Unreal game engine, developed by the WANDER Lab at Wageningen University and Research.

### Testing approach

Testing took place at the University of Aberdeen, with full ethical approval for the use of a HMD, EDA sensors and post-survey assessment (ethical approval project number: SERB/2022/4/2333—Testing XR Technology for Teaching Biochemical Processes). The sample size was 10 volunteer students recruited through advertisement of the research to students on Medical Science undergraduate degree programmes. All testing was conducted in a large area free of furniture to allow players to move freely. As Van Voorhis and Morgan et al*.* outline, for regression-based assessment (with > 6 predictor variables), one should have a minimum of *n* = *10* participants (vanVoorhis & Morgan, 2007) given an expected normal distribution and a large effect size.

The recruitment announcement provided them with the opportunity to review a Participant Information Sheet (PIS), at least one week prior to taking part in the study. Once recruited, students were assigned a specific day to meet at an agreed teaching venue, where they are given another opportunity to review the PIS and provided with consent forms for reading and signing. Students were allocated a unique study ID (not known to them) and given time to familiarise themselves with the technology being used with the aid of an instructor to answer any queries they may have.

Participants were then permitted to interact with the VR visualisations until they wished to stop. It should be noted that all participants completed the eight levels in the VR environment and, as such, interacted with the VR to completion of the exercise. Interaction involved using VR HMD placed over the eyes and a hand-held controller to navigate and move around the virtual environment. During the interactions with the visualisations, physiological measurements of skin conductance and heart rate were recorded via an Empatica E4 wristband device to measure engagement. There was no minimum time for these measurements to be recorded as the devices were switched on as participants placed them around their wrists. Timestamps were added into the dataset by asking participants to push the button on the wristband to add events into the recording. This allowed for synchronisation of events in the recording and events occurring in the VR (e.g., completion of a level). All engagement recordings were taken automatically and non-invasively so other than creating timestamps, participants did not need to interact with the wristband device during the session. Thus, in Section 4, electrodermal activity results will be analysed for changes in EDA response rather than comparisons of actual electrodermal activity between individuals to account for differences in individual participants.

### Survey approach

Following the session, participants were asked to complete an online survey and a focus group session. The classroom activity lasted for varying amounts of time for each individual, the survey lasted for around 10 min per individual and the focus group ran for approximately 30 min with breaks in between. As with the physiological data, all survey data was collected anonymously. Focus groups also involved voice recordings being taken, which were transcribed with all comments anonymised prior to analysis. A summary of the survey approach is presented in Table 9 in the Appendix.

Ultimately the intention of the surveys and interviews was to gain more understanding of how students perceive the use of this type of technology in education using the various levels of the Kirkpatrick model of engagement. The four levels of the model are, *Level 1*: Students’ reaction to the session, *Level 2*: Students’ ability to articulate learning, *Level 3*: Students’ behaviour post-session and *Level 4*: Benefits derived from the session (Partners & “What Is The Kirkpatrick Model”, 2022).

### Data evaluation approach

The data evaluation approach was poised around a multiple linear regression, performed in RStudio, where the *car* and *lmtest* libraries were used for the experimentation. Multiple linear regression is within the supervised machine learning domain, involving calculation of a best fitting line from multiple predictor variables, and is used as a measure of statistical significance, from which p and t values are considered. A p-value of < 0.05 and a t-test value of > 2 is conventionally defined as the threshold for statistical significance. The algorithm can be expressed in matrix form as follows in (1), derived from Singh et al. (2021).

1 $$Y = X\beta + \varepsilon$$

Further, data point distance from the line of regression is known as residual error, and is denoted by the MSE Eq. (2) (Singh et al., 2021)

2 $$MSE = \frac{1}{n}{\sum }_{i=1}^{n}{\left({y}_{i}-{\widehat{y}}_{i}\right)}^{2}$$

We also assessed the performance by means of both Diagnostic and Quantile–Quantile (Q-Q) plots for visual inspection of outliers in the survey responses. A Diagnostic plot presents three sets of results: 1) Cook’s Distance, which is the influence values; 2) Studentized, which is a visualisation of outliers; and 3) Hat, which is the leverage. A Cook’s distance (d-value) of > 1 is indicative of influence.

The intention is that any clear outliers can be visually inspected in the Studentized graph. The Q-Q plot displays a comparison of two probability distributions. The intention of the multiple linear regression approach is to statistically link how responses to (online survey) questions 3 to 9 link to the students’ overall feelings about their increase in learning or relevance of the technology for learning (questions 1 and 2 – see Table 1).

**Table 1:** Post-VR Individual Survey Questions

Question	Full Question
Q1	The VR visualisations have increased my understanding
Q2	I feel the VR visualisations were relevant to my learning
Q3	I felt more engaged using VR technology compared to being in a lecture
Q4	I felt more engaged using VR technology compared to reading a textbook
Q5	I feel that the use of VR technology has stimulated my interest in the subjects covered
Q6	I feel more confident in my understanding of the subjects covered after using VR technology
Q7	It would be useful for me to have this method of learning available to support my learning of other biochemical or cellular process
Q8	I am likely to use this type of technology again when revising for future assessments on these topics
Q9	Overall, my time using the XR technology was useful to my learning

## Results

User trials took place in two different sessions in October 2022 at the University of Aberdeen. In each session, 5 participants took part in testing the VR application. In this section an overview of the survey data is first provided, followed by the Kirkpatrick evaluation, statistical analysis, and presentation of the EDA findings.

### Data overview

**Fig. 5:**
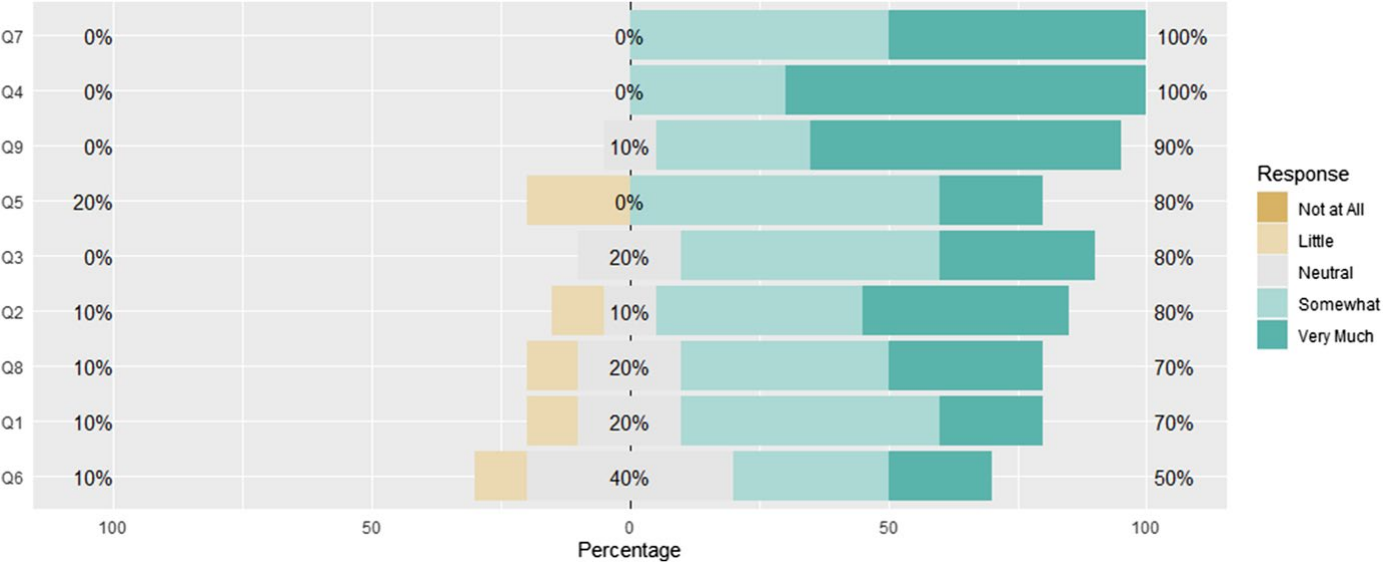
Likert Plot for 10 Participants ordered by Positive to Negative along the Y-Axis

For the online survey, all 10 participants responded, in an average time of 17 min 51 s. Table 1 displays the list of first 9 questions asked, and the corresponding abbreviation (Q1:Q9). All participants are between ages 18 and 34, 6 of whom are in their 3^rd^ year of undergraduate education and 4 in their 4^th^ year. A Likert plot in Fig. 5 provides a visual overview of the questions responses, ordered by weight of positive to negative on the Y-Axis.

For example, Q7: ‘It would be useful for me to have this method of learning available to support my learning of other biochemical or cellular process’, had a 100% positive feedback score (i.e. students selected either 4 or 5 in the Likert scale), whereas Q6: ‘I feel more confident in my understanding of the subjects covered after using XR technology’ had more of a mixed result, with 40% of students feeling more neutral (and providing a score of 3).

### Kirkpatrick & kirkpatrick evaluation

To further analyse the data from the survey the Kirkpatrick model of analysis was adopted, which is an internationally recognised method to assess the effectiveness of training and learning activities through four levels of evaluation: Reaction, Learning, Behaviour and Results. The questions posed in the online survey and those in the focus groups align to this model as shown in Table 2.

**Table 2:** Kirkpatrick & Kirkpatrick Evaluation Questions Overview

K&K Level	Survey Questions	Focus Group Questions
Reaction	Q1-Q9 (Immediate Post-VR Likert Questions—see Table 1)	FQ1—Thinking back to using the VR technology, tell me your initial reaction to using it?
	Q10—What did you like best about the VR technology?	FQ2—What did you like the most about the session?
	Q11—What did you like least about the VR technology?	FQ3—What did you like least about the session?
Learning	Q12—Before using VR technology in today's session, I would rate my understanding of the topics covered as:	FQ4—The session was centred around using XR technologies to visualise molecular processes, so do you think this worked for you?
	Q13—After using VR technology in today's session, I would rate my understanding of the topics covered as:	FQ5—Tell me about the knowledge you think you have developed by visualising processes in this way?
	Q14—In your own words, please describe what you have learned about the topics covered using VR technology	
	Q15—Please list three words or phrases you would use to describe VR technology in education	
Behaviour	Q16—In your own words, please describe what aspects of the VR technology you would like to have a better understanding of after using it today	FQ6—Has your thinking changed in any way regarding biochemistry/molecular biology following the session?
	Q17—In which fields other than biochemistry could you see a similar approach being useful for teaching difficult or abstract concepts?	
Results	Q18—What other types of technology have been used to support your studies so far?	FQ7—Do you think this will work for other disciplines or subjects?
	Q19—What other types of visualisation approaches to teaching or learning would you like to see taken to support your studies?	
	Q20—Do you think VR visualisations are a useful tool in education? Why?	

Thematic analysis of the free text responses from participants responses to Q10 and Q11 in the survey on what they liked best and least when using the VR technology produced five themes. Educational and Engaging are only seen as positive reactions to the use of VR by 4 and 5 respondents respectively. The theme of Interactive gives a higher proportion of positive responses with 6 positive and 2 negative comments. The themes of Physical and Visual produces a more comparable number of positive and negative responses with only a difference of 1 respondent in both themes. The focus group data indicates similar responses with the educational and engaging aspects of VR being seen as very positive and the ability to interact, physically move and the visualisation proving less positive for many participants is often a negative aspect. Further, visual overload and controllability of the game components are highlighted in the discussion.

From the survey, participants are asked to rate their understanding of the topics covered in the VR session from 1 (Very Low) to 5 (Very High). On average there is an increase of 1 Likert scale point following the VR session (see Table 3.).

**Table 3:** Likert scale scoring based on the statement “Before/After using VR technology in today's session, I would rate my understanding of the topics covered as:”

User	Before	After	Gain
1	2	3	1
2	4	5	1
3	3	4	1
4	3	4	1
5	3	4	1
6	3	3	0
7	4	5	1
8	3	5	2
9	3	4	1
10	2	3	1

This is reinforced in the free text survey question asking participants to describe what they have learned about the topics covered in the VR session (Survey Q14), which sees responses that are all positive in nature and highlighted areas that students had understood better after the VR session; such as “*The VR technology has reinforced knowledge that was learnt in last year’s Energy of Life course*” and “*I have now understood more which molecules interact with which enzyme and in which order. I also now feel like I remember more of the molecules (and names) which take part in the reactions*”.

The survey also asks participants to list their top 3 words that describe using VR technology for visualising biochemical processes. Word cloud analysis of this data shows the top word is interactive. With visual learning, innovative and immersive the second most common words used to describe the experience. This is supported by commentary from the focus group session, especially for visualisation and enhancing understanding, as two themes that stood out in conversation with participants (see Q4 and Q5 in Table 2).

Participants are also asked to describe the aspects of VR that they would like to understand more about and what other subjects this method might work well in (Survey Q16 and Q17 respectively). Overall, a relatively even split of answers to Q16 with responses being coded and showing the themes of future developments, additional functionality, technical and educational. This highlights that students are responsive to VR, in that they would like to see more developments in this area going forward. Further, they thought having added functions within the VR learning environment would work well. They also hold the view that this technology could be pushed technically beyond that of the processes and visualisations they had used in the VR experience of this trial, and they saw a great potential using this type of visualisation technology in education (see Table 4).

**Table 4:** Participants’ view of educational benefits of VR

Question	Response	Percentage (%)
Aspects of VR participants would like to have a better understanding of following the session	Future Developments Additional Functionality Technical Educational	28 27 18 27
Disciplines where VR would work well	Mol. Bio Anatomy Physiology Cells Pharm Chem Dev. Biol Other	12 17 4 13 4 13 4 33

**Fig. 6:**
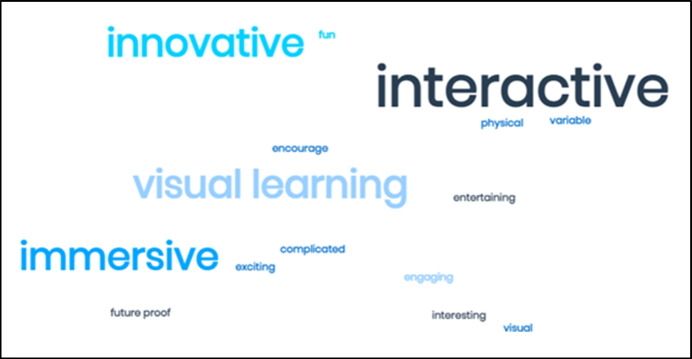
Word cloud analysis of Survey Q15

For the final Results’ level of the Kirkpatrick analysis (via the survey questions 18–20) participants highlight that they use various forms of technology that could be themed into Visualisation, Gamification, Online Education, Simulations, and Lab Equipment. The largest proportion of responses to Survey Q18 is the use of technology to support online education, which is perhaps not surprising following the pivot to online or blended modes of delivery due to the Covid-19 global pandemic. When asked about the types of technology that participants would like to see in their education the biggest proportion of coded responses is in the theme of Interactive. This mirrors responses on what students felt were the most positive aspects of the VR experience for students (Fig. 6.).

Finally, the thematic analysis of responses to Survey Q20 clearly shows that the perceived benefits of the use of VR in education as being relatively evenly split between Simplification, Interactivity, Impossible, and Memorable, with very few responses being about Enjoyment (see Table 5). In this context, Impossible means that they would like to see visualisations that are impossible to visualise in another way because the scene is impossible to recreate. Further to the survey questions in the focus group analysis, the data shows multiple examples of ways that VR could be used in other areas (see Tables 5 and 6).

**Table 5:** Focus Group Assessment of Ways that VR Could be Used in Other Areas

Question	Response	Percentage (%)
Types of technology already used to support studies of participants	Visualisation Gamification Online Education Simulations Lab Equipment	20 7 33 13 27
Types of technology participants would like to see in supporting their education	Interactive Processes Concepts Training	37 25 13 25
Themes from the usefulness of VR technology	Enjoyment Simplification Interactivity Impossible Memorable	6 22 22 22 28

**Table 6:** Sample of Verbal Responses in Focus Group, Organised by Theme

Focus Group Questions	Theme	Quote
*FQ1 / FQ2 / FQ3*	*Positive*	“So physically being able to handle [items]” “I like the sort of interactivity as well and that you could do three steps, then go fool around a little bit and then go through the next three steps that you didn't have to do it all in one go. And you had that sort of like time in between.”
	*Negative*	“…the darkness, just because at multiple times I wanted to take it off…There's this fright element that affected me…I really disconnected from everything I wanted to take it off.” “…it was just a bit confusing at the start because I didn't know how to like control stuff and it cannot get to, you know, you're like, OK, what is this? How do I use this?”
*FQ4*	*Visualisation*	“I think that visualisation is the best way to understand these concepts.” “Even if you don't try to kind of think in the visualisation part, the VR switches on the visualisation automatically inside of you.”
*FQ5*	*Understanding*	“…the protein shapes really stuck out to me…then you see how big the enzyme is. Is it big? Is it small? You get a much better understanding of what kind of conversion is going to happen.”
*FQ6*	*Behaviour change*	“I wonder why I haven't looked at any animations before…I'm making a mental promise to myself to integrate [visualisations] next time I'm studying [to] probably make a big difference.”
*FQ7*	*Impossible*	“Geoscience. Right? You can go through the layers of the mantle and the crust and deep down into the earth.”
*FQ8*	*Interactivity*	“I went into the degree with little knowledge of what academia means, etc. for different reasons. And I share that this was something that a lot of people didn't have this vision of what to expect, what kind of pathways. And I can vividly imagine that in this VR, for example, gamification of the process of learning or the targets to meet or like a room of expectations.”

### Statistical assessment

The focus of the statistical analysis is on both Q1 ‘*The XR visualisations have increased my understanding*’ and Q2 ‘*I feel the XR visualisations were relevant to my learning. *(Table 2)’ as, in each, the attention is directly on the students’ learning opinion. Thus, two analyses were conducted, in which Q3:Q9 serve as the predictor variables. Table 7 displays the scores for Estimate, Standard Error, *t*- and p-values, with Q1 as the outcome variable. In this analysis, the residual standard error is low at 0.21, the multiple R-squared outcome is 0.988 (with an adjusted R-squared of 0.946) and an *F*-statistic of 23.62. The overall *p*-value is 0.041, and reaches the significant threshold of *p* < 0.05. As mentioned earlier, it is assumed for the analysis that the selected sample is normally distributed. Furthermore, based on previous research indicating that the perceived benefits of VR in education are large (Huang et al., 2019), it was assumed that the effect size is high. However, given the relatively small sample size, questions may be raised about statistical power of our analysis. These issues that are discussed in the limitations and future research section.

**Table 7:** Multiple Linear Regression Scores for Standard Error, t and p values for Q1

Question	Std. Error	t value	Pr( >|t|)
Q3	0.3072	-6.201	0.025
Q4	0.4464	0.712	0.550
Q5	0.2189	4.858	0.040
Q6	0.2470	2.238	0.155
Q7	0.3892	3.280	0.082
Q8	0.1969	6.401	0.024
Q9	0.3012	-4.596	0.044

**Fig. 7:**
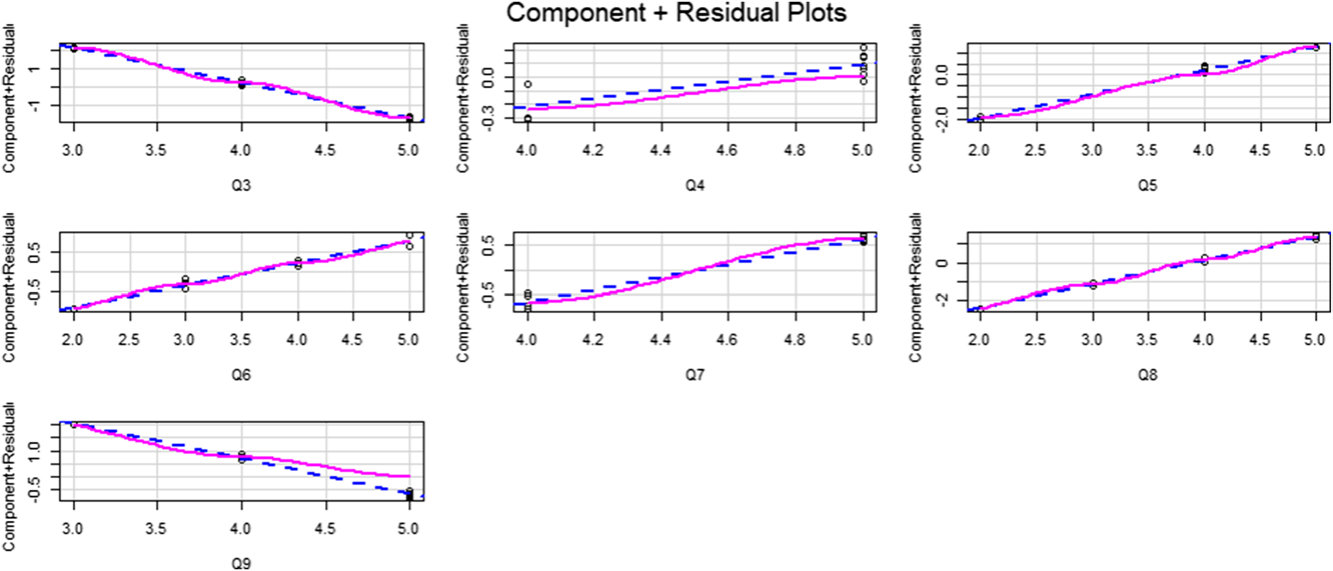
Component and Residual Scores for Q1

Results indicate that Q3 (I felt more engaged using XR technology compared to being in a lecture.), Q5 (I feel that the use of XR technology has stimulated my interest in the subjects covered.), Q8 (I am likely to use this type of technology again when revising for future assessments on these topics.) and Q9 (Overall, my time using the XR technology was useful to my learning.) are statistically significant. Component and residual scores are displayed in the Q-Q plot in Fig. 7, where the blue line represents where the linear relationship should be.

**Fig. 8:**
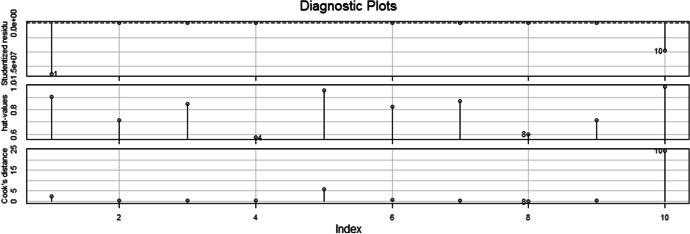
Diagnostics Plot for Q1

Figure 8 displays a Diagnostic plot, with the 10 participants displayed on the x-axis, where the Studentized error, hat-value and cook’s distance are indicated on the y-axis. In this instance, the Studentized error refers to outliers, which can be visually inspected.

The influence values are denoted by a Cook’s distance (*d*-value), where a value > 1 is indicative of influence. Leverage (hat) is also indicated. From the Studentized error and Cook’s distance, we see that User 10 is an outlier (which is later investigated after experiment 2). In summary, if the students feel engaged, stimulated, intend to use the technology again and find it useful, they will be likely to say that the VR has increased their understanding.

For the assessment of Q2, no statistically link between the predictor variables (Q3:Q9) and the outcome variable is found. Results from the experiment are outlined in Table 8. Further, the Residual standard error was quite high at 0.526, with a Multiple R-squared of 0.9379 (and Adjusted R-squared of 0.7204). The *F*-statistic is 4.313, with a *p*-value of 0.2011, well above the 0.05 threshold.

**Table 8:** Multiple Linear Regression Scores for Standard Error, t and p values for Q2

Question	Std. Error	t value	Pr( >|t|)
Q3	0.7581	0.367	0.749
Q4	1.1014	-1.625	0.246
Q5	0.5402	0.818	0.499
Q6	0.6096	2.245	0.154
Q7	0.9604	1.754	0.222
Q8	0.4858	-1.273	0.331
Q9	0.7432	0.468	0.686

**Fig. 9:**
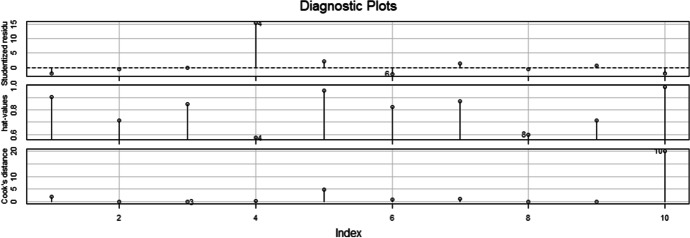
Diagnostics Plot for Q2

As mentioned, User 10 is an outlier, and this is the case for both experiments as also evident in Fig. 9. On further inspection of the survey data, this could be due to the user providing conflicting scores in the survey. For example, the individual selected 5/5 for Q10 ‘*Overall, my time using the VR technology was useful to my learning.*’ demonstrating that they felt that the VR application is very much useful for their learning. Yet, for other questions related to confidence and increased understanding, they provided negative responses, including a Likert score of 2/5 for Q7 ‘*I feel more confident in my understanding of the subjects covered after using XR technology*.’ and 2/5 for Q1 ‘*The XR visualisations have increased my understanding*’. The individual also responded negatively when asked if they would like to use VR technologies again for learning, also providing a score of 2/5. This confliction may have resulted in the high outlier value.

### Physiological data insight

As mentioned, EDA measured in microsiemens (µS) is a potential measure of physiological arousal (i.e., engagement or excitement) in a given activity. Inspection of the raw EDA signal output from the Empatica E4 wristband indicates that many of the participants show an increase in EDA signal, which signifies an enhanced level of excitement or engagement. Some participants showed considerable marked increases in their EDA signal from the beginning to the end of the session and nearly all participants showed a spike in their EDA signal in the final few moments of the session; potentially indicating that the completion of the VR task enhanced their excitement even further as they completed all eight levels of the VR experience. It should also be noted that the total time spent in the VR experience varied between individuals as some carried out the tasks required quickly and others took more time. Specifically, the variation is from a minimum of 6 min (Participant I) to a maximum of 23 min (Participant A) with an average time of 12.57 ± SD 5.9 min.

An overall decrease in time spent in VR (y = -1.3981x + 20.26, R^2^ = 0.5135) is observed when progressing through the candidates. This is likely accredited to future participants observing previous users operating the VR headset, teaching them how to use the headset more efficiently. The trend repeated itself between participant groups. Where A-E (y = -2.497x + 22.819, R^2^ = 0.503) and F-J (y = -2.147x + 16.257, R^2^ = 0.4038) both show a decrease in time spent in VR over participant number. We suggest that this can be leveraged in education, where allowing students to observe their peers in VR would decrease overall time.

**Fig. 10:**
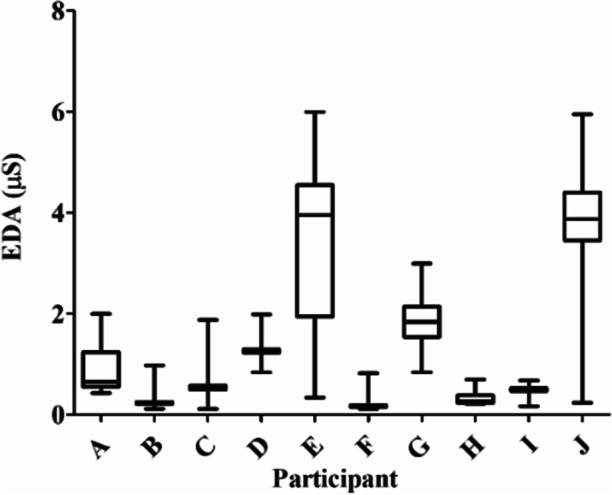
Box-Whisker plot of raw EMG signals from the E4 wristband recorded throughout the VR session for each participant

To assess the variability between data sets an initial Box-Whisker plot was constructed (Fig. 10), showing EDA variability for all participants throughout the VR experience 0.11–5.99 μS with baseline variability established as 0.24–0.97μS. Main factors affecting EDA variability can be categorised as participant specific and environmental. Participant specific factors concern baseline sweat gland activity, sebum secretion and levels of electrolytes present on the skin surface (Bake & Wolfe, 2020).

Environmental factors mainly concern the activity levels of the participant before arriving to the study, especially physically strenuous activities such as biking or walking. Participants E and J, being the last participants of their respective cohorts are noted to have arrived late to the study. Thus, to arrive on time, E and J most likely exerted themselves physically before attending the study as reflected in their high EDA variability (E = 5.66 μS; J = 5.71 μS). Additionally, the mean EDA of E and J (3.64 μS) is notably higher than the rest of the cohort (0.71μS). Temporally participants E and J fall within the 25-min recovery phase after stress conditions (Setz et al., 2010), suggesting that the raised average EDA and increased variability originated from physical exertion.

**Fig. 11:**
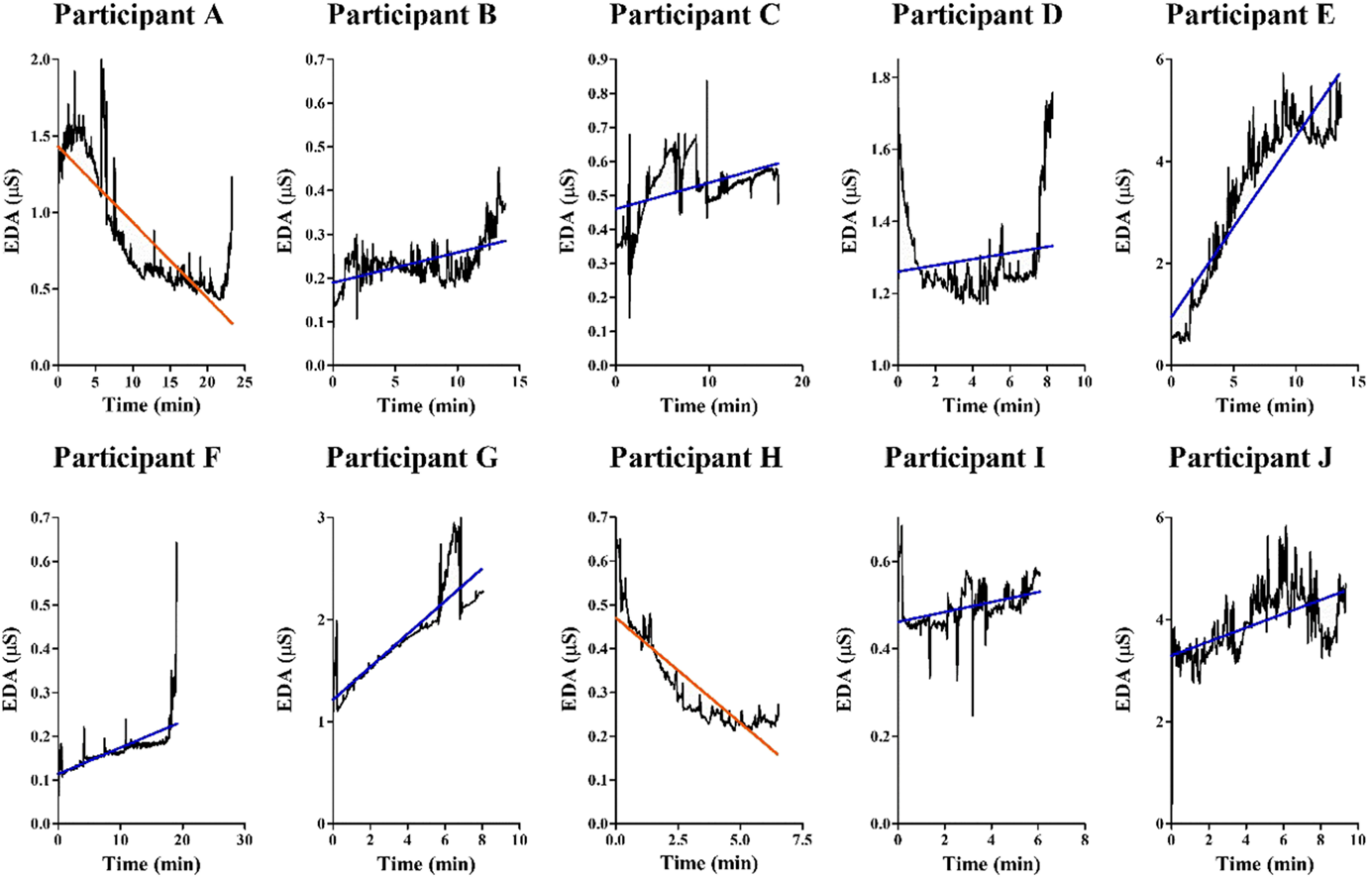
Smoothed scatterplot of EDA against time in VR experience in milliseconds. Linear regression line of best fit based on non smoothed data added, coloured based on overall increase (Blue) or decrease (Orange) in EDA

As EDA can be divided into tonic and phasic changes, with phasic changes appearing as a direct response to stimulus, whilst tonic outline the general arousal level of the participant. We assessed the tonic EDA response to VR separately from the phasic response. To observe if a trend of gain or loss in EDA signal is observed, a linear regression line was plotted using the data of each participant. The data was subsequently cleaned using neighbour smoothing and polynomial available in Prism 9.0. Figure 11 displays the combed unmodified linear regression line with the scatterplot of cleaned EDA signal plotted against time.

Participants showing an overall increase in EDA over time are shown with a blue linear regression line, and overall decreases in EDA are shown in orange. For all participants except A and H an overall increase in tonic EDA signal can be observed. This data supports the previously suggested link between an education-based VR-experience and an increase in arousal markers. On average the participants experiencing hypothesised arousal had an increase in EDA signal of 0.086 μS/min. In contrast, participants not experiencing arousal have a decrease in EDA signal of 0.025 μS/min. Suggesting that when positively experienced, VR education has the potential to make students more engaged and subsequently more involved in the task. We hypothesise that the decrease in arousal in participants A and H can be attributed to stress or potentially due to individual biological differences.

With participant A being the first one undertaking the study they might have been anxious or felt insecure about the task. As for participant H we note probable nyctophobia. The participant expressed their dislike for dark environments during the group discussion, highlighting that the transition between levels, where the screen became partially dark, made them uncomfortable. An increase in anxiety levels has previously been established to increase sweat production (Nikolić et al., 2017), and suggests that if these participants were anxious an increase in EDA similar to aroused participants should be present.

**Fig. 12:**
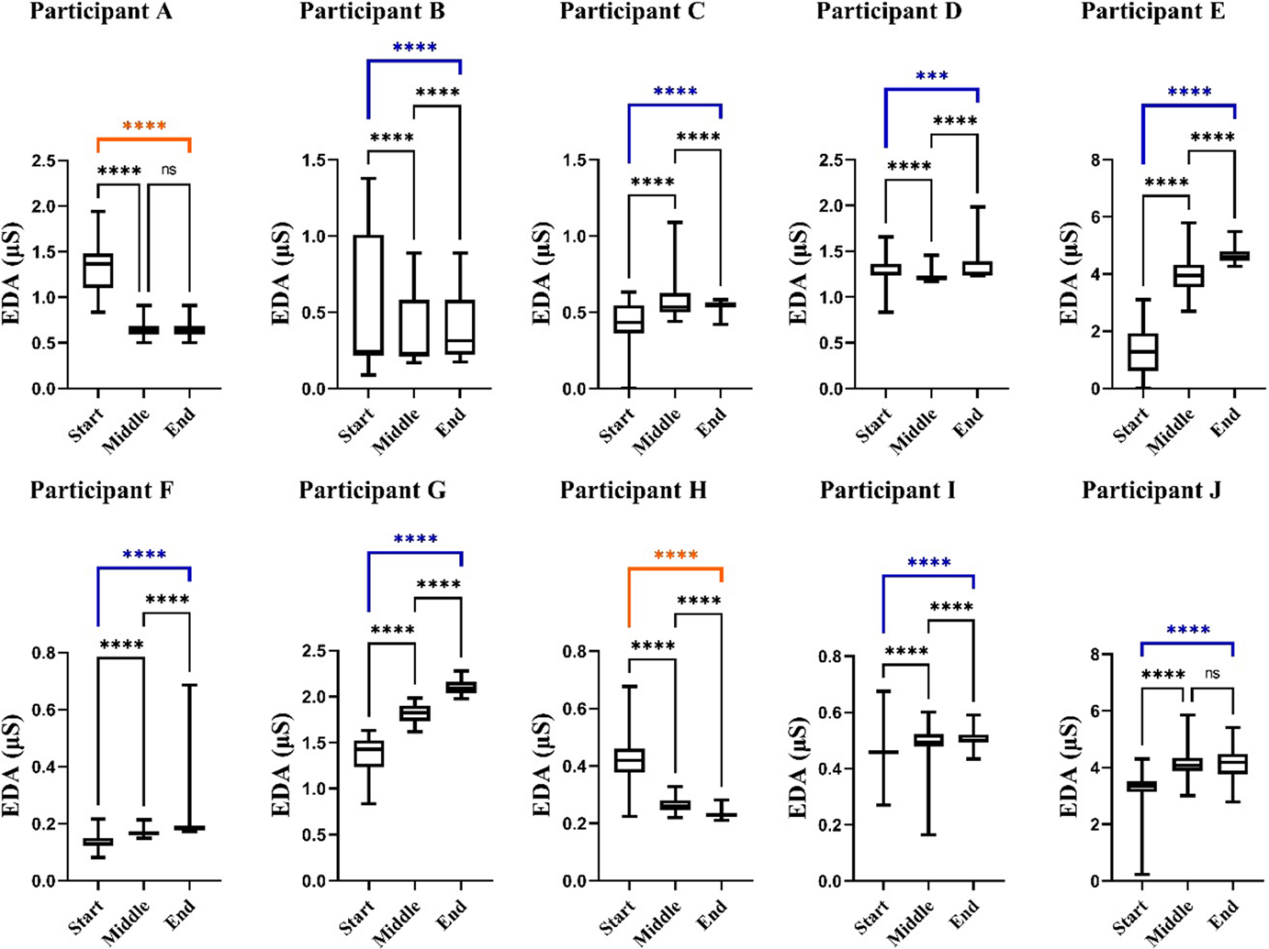
Box-Whisker plot of raw EMG output from the E4 wristband recorded throughout the VR session for each participant, data has been separated into three equal groups for each participant (start, middle and end). Significance brackets added, obtained from performed non-parametric Friedmann test in Prism 9.0. Colours signify increase (Blue) or decrease (Orange) in EDA between start and end thirds

However, during parasympathetic overactivation occurring during a perceived stressful time period secondary sweat glands (apocrine) activate, secreting sweat of different composition (Chen et al., 2020). The impact of apocrine vs. eccrine sweat on EDA is not able to be assessed in the scope of this study. To assess if the differences between starting, middle and ending tonic EDA are significant, a Friedmann test was performed between data groups for each participant. For the scatterplots, colours are assigned to indicate overall decrease (orange) or increase (blue) in EDA (Fig. 12).

For all participants a significant change in EDA is observed between the start and end of the VR-experience. Notably, a significant change was globally observed between start and middle, compared to lack of significance between middle and end for participants A and J. Suggesting that the arousal, created by the VR experience is stronger at the start of the experience than at the end. With the tonic EDA signals previously analysed, phasic signals in direct response to progression in the game were also analysed. All participants are asked to indicate completion of a level by pressing the button on the Empatica E4 wristband. For many participants this was a challenge with two VR handsets and many cited engagement in the experience as the reason they forgot to press the button. However, one participant (Participant H) successfully managed to complete this task. An average increase in EDA of 0.03 μS is noted for participant H, occurring 5–10 s before pressing the button to mark completion of each level in the VR experience (data not shown).

As only one sample showing clear phasic EDA signals was obtained, no conclusions can be made on a study level. However, we suggest that in response to completing a level an initial wave of excitement reflected in the EDA output can be observed.

### Limitations and future research suggestions

Even if VR technology has demonstrated to be a valuable tool in education, we acknowledge the limitations coupled to widespread uptake of this approach. Amongst these, visually induced motion sickness (VIMS) is a key limiting factor. Occurring as a result of mismatched vestibular and visual information integration, this leads to the person experiencing nausea and disorientation (Golding, 2016). The key conflict arises as a result of perceived visual movement (vection) in contrast to perceived vestibular lack of movement (stasis). Although VIMS has been a known issue since its characterization by Hettinger & Riccio in 1992, few credible implementations to reduce it have been suggested (Hettinger & Riccio, 1992). Amongst these suggestions the implementation of static and dynamic rest frames in the VR environment is a cost-effective way to reduce VIMS (Cao et al., 2018). This method, defined by regions of the frame that stay constant throughout the experience work similarly to the horizon-line effect, reducing overall motion sickness (Horizon, 2020). Furthermore, human perception favours the observation of stationary objects to provide a reference point, a feature that multiple VR environments lack. Hence, we suggest that the implementation of dynamic rest frames in VR environments targeted for educational purposes can reduce VIMS significantly.

Focusing on VR in education, the issue of VIMS is further highlighted. As shown by Bertolini & Straumann, increasing the spatial complexity of the environment positively correlates with the likelihood motion sickness (Bertolini & Straumann, 2016). Furthermore, the cognitive decline observed as a result of VIMS is proportional to the complexity of the task undertaken in VR (Gresty & Golding, 2009). In our case, the teaching of biochemical processes using VR, which relies on displaying complex molecules and interactions, thus strengthens the need for a VIMS reducing component. Another solution, not feasible to be implemented in large scale education, is the concept of motion sickness reduction through repeat exposure to the environment (Gavgani et al., 2016). This approach shows game- and time-specific trends, where simpler games with increased exposure time decrease symptoms the most (Palmisano & Constable, 2022). As time constraints are a key component of teaching, this approach is suggested to be implemented in cases where group size is small and time constraints are not present. Alternatively, a VR course that allows students to be exposed to VR multiple times before teaching is suggested.

Interestingly, the severity of VIMS symptoms also follows gender and eyesight trends. VIMS has an increased prevalence in females, accredited to smaller average interpupillary distance (IPD) (Osuobeni & Al-Musa, 1993; Stanney et al., 2020). The lack of adjustability for low IPD, as determined by the manufacturer, is the suggested cause. Similarly, students with impaired eyesight are more likely to require a broader IPD adjustment range to be able to focus on objects in the VR environment. As we aim to create a teaching environment which is inclusive to all students these factors need to be accounted for, preferentially selecting VR displays with a broad range of IPD adjustability.

Further, the VR-based benefits for learning demonstrated in this article can be considered domain specific and one specific application scenario. Further testing should be conducted for other complex educational material to offer additional evidence that the approach is suitable for other domains. This could also include validation of the approach, using field work experiments in which student use different learning approaches such as VR vs. non VR. There is also risk of bias in the findings, as students are volunteers, which may indicate an existing interest/affinity for VR-based technologies meaning a greater likelihood for positive feedback. Furthermore, supported by the results of measured EDA arousal, which decreases over time, VR in education might have a *novelty effect* when students use VR for the first time. Longitudinal studies need to examine the initial findings to investigate whether they are long-lasting, valid effects. Our approach to mitigate this risk was to avoid use of any questions related to enjoyment of the application, but rather a focus on engagement, learning and likelihood (to use or reuse VR for learning), as reflected in Q1:Q9. It should, however, be noted that the survey results are based on the assumption of an normal distributed sample size and high effect size, aspects that cannot be fully verified in this study. Therefore, larger sample sizes will be needed in future research to validate the initial statistical results of this study. As a final though, despite the growing volume of examples of successful VR deployment with biochemistry education, development of VR-based learning environments still requires a substantial technical background. SDKs and hardware affordability are currently reducing this requirement, but at present, complex implementation still currently requires technical serious game development skills.

## Conclusion and future work

In this article, a VR application of the citric acid cycle is presented, in which students were able to find an immersive and alternate approach to traditional book-based learning of the complex subject matter. From the survey analysis, involving both online survey and focus group session, the student participants were predominantly positive about the learning experience, and by means of the Kirkpatrick evaluation framework a gain in understanding of the subject material seen by 9 of the 10 participants is evident. Multiple Linear Regression findings support the hypothesis that VR increase students’ understanding, particularly if students feel engaged, stimulated and intend to use the technology. Further, when employing EDA measurement (as a measure of potential physiological arousal) findings show that when positively experienced, VR education has the potential to make students more aroused and subsequently more involved in the task they are conducting.

## Data Availability

The datasets generated during and/or analysed during the current study are not publicly available due the ethical constraints regarding the sharing of personal data but samples of the anonymised data can be made available from the corresponding author on reasonable request.

## Appendix

Table 9.

**Table 9:** Survey Approach Summary

Item	Description
*Sign-up sheet data*	Participants were recruited by completing a sign-up sheet via Microsoft Forms. This data was collected prior to consent and asked for their name, email address, and student ID so that they could be contacted and verified as being a student at the University respectively. Once this data was used to recruit individuals to the study, the information as permanently deleted so all subsequent data (see below) was captured anonymously. Data collected in the sign-up sheet was not linked to the physiological measurements, survey or focus group data
*Cognitive engagement data*	Following completion of the visualisation activities, participants were asked to remove the wristband device and the data was downloaded for analysis. All data downloaded was stored on a password protected network drive on the University network and identified through a simple numbering system (i.e. Participant A, Participant B, etc.)
*Survey and interview data*	Survey data was saved to the Office 365 account of the principal investigator and password protected. Any downloaded data from this survey was saved to a password protected network drive alongside all other data for this project. Interview recordings were taken using a digital recording device and, immediately following the interviews, downloaded and saved to the same network drive as all other project data. The original recordings were then deleted from the recording device. Interview recordings were auto-transcribed through NVivo software and the transcripts proof-read to check for accuracy by the investigators
*Physical forms*	The only physical paperwork that was stored was participant consent forms which were stored securely in a locked filing cabinet following completion of the forms by each participant. The consent form mentions non-identifiable data being stored on secure computers
